# *Escherichia coli* Cluster Evaluation

**DOI:** 10.3201/eid1303.041085

**Published:** 2007-03

**Authors:** James R. Johnson

**Affiliations:** * University of Minnesota, Minneapolis, Minnesota, USA

**Keywords:** clone, genetic similarity, pulsed-field gel electrophoresis, Escherichia coli, phylogenetic analysis, outbreak, molecular epidemiology, letter

**To the Editor:** Gupta et al. raise important issues regarding molecular profiling as an epidemiologic tool (*1*). First, since all living organisms are related, the goal of genomic profiling in public health epidemiology is not really to determine “whether such isolates are truly related” (*1*) (they are), but to define the degree of similarity—or, more specifically, to determine whether isolates are sufficiently closely related that the probability of their deriving immediately from the same point source is high enough to warrant epidemiologic investigation. Second, definitive assessment of genetic similarity relationships is challenging because of the limited accuracy and resolving power of conventional methods such as pulsed-field gel electrophoresis (PFGE) analysis (*2*) and the impracticality and expense of better performing technologies. Sequential use of multiple methods (such as PFGE with additional restriction enzymes) will predictably detect additional differences, thereby improving resolving power (*2*). Third, even if genetic similarity could be precisely defined, the relationship between the degree of genetic similarity and the probability of point-source spread is unknown and doubtless varies in relation to pretest probability, depending on the epidemiologic context (e.g., localized vs. multistate clusters). Even <100% similarity may be compatible with point-source spread when genetic drift exists within the reservoir, leading to dissemination of highly similar but nonidentical clones.

Gupta et al. interpret their experience as indicating that, with geographically dispersed isolates, a higher degree of genomic similarity than is reliably provided by single-enzyme PFGE is necessary to improve specificity, thereby avoiding fruitless investigative efforts (*1*). However, whether the subclusters shown by their second-round PFGE were more epidemiologically meaningful than the original cluster remains unclear, nor do we know how representative this experience is. Determination of optimal genetic similarity parameters for geographically distributed epidemiologic surveillance (e.g., through PulseNet) would seem to require more in-depth empirical assessment, possibly incorporating Bayesian likelihood (*3*).
